# Experimental Study on Mechanical and Functional Properties of Reduced Graphene Oxide/Cement Composites

**DOI:** 10.3390/ma13133015

**Published:** 2020-07-06

**Authors:** Ning Zhang, Wei She, Fengyin Du, Kaili Xu

**Affiliations:** 1 School of Mechanical Engineering, Southeast University, Nanjing 211189, China; nzhang_cn@seu.edu.cn; 2 Jiangsu Key Laboratory for Construction Materials, Southeast University, Nanjing 211189, China; yzdufengyin@126.com; 3 Shandong Institute for Product Quality Inspection, Jinan 250100, China; 18351959226@163.com

**Keywords:** reduced graphene oxide, cement-based materials, electrical properties, pressure sensitivity, electromagnetic shielding properties

## Abstract

This study develops a novel self-sensing cement composite by simply mixing reduced graphene oxide (rGO) in cementitious material. The experimental results indicate that, owing to the excellent dispersion method, the nucleation and two-dimensional morphological effect of rGO optimizes the microstructure inside cement-based material. This would increase the electric conductivity, thermal property and self-induction system of cement material, making it much easier for cementitious material to better warn about impending damage. The use of rGO can improve the electric conductivity and electric shielding property of rGO-paste by 23% and 45%. The remarkable enhancement was that the voltage change rate of 1.00 wt.%-rGO paste under six-cycle loads increased from 4% to 12.6%, with strain sensitivity up to 363.10, without compromising the mechanical properties. The maximum compressive strength of the rGO-mortar can be increased from 55 MPa to 71 MPa. In conclusion, the research findings provide an effective strategy to functionalize cement materials by mixing rGO and to achieve the stronger electric shielding property and higher-pressure sensitivity of rGO–cement composites, leading to the development of a novel high strength self-sensing cement material with a flexural strength up to 49%.

## 1. Introduction

Cement-based composites are the most widely used construction materials in the world, and the bearing of various loads or gradual erosion from environments are inevitable [1,2,3]. However, cement-based composite is a kind of brittle material and is susceptible to cracking. Once cracked, the cracks will provide an easy way for the ingress of aggressive ions, which will further threaten the safety of the infrastructure [4,5]. Therefore, it is important and urgent to improve the mechanical properties of cement-based materials and provide regular monitoring to ensure safety of cement concrete composites [6].

Graphene, as a new two-dimensional nanomaterial, has excellent mechanical properties. The tensile strength exceeds 50 GPa and the Young′s modulus is about 1 TPa [7]. The electron mobility of graphene is as high as 2.5 × 10^5^ cm^2^/(V s) [8] at room temperature and its thermal conductivity is up to 5300 W/(m K) [9], much higher than carbon nanotubes and diamond. If graphene is incorporated into cement materials, it is easy to disperse in the cement matrix to form a network structure, make the internal structure dense and it plays a role of strengthening and toughening, due to the nanometer size and two-dimensional morphological effects of graphene [9]. At the same time, its excellent thermal and electrical conductivity improve certain functionalities of cement-based materials [10,11]. Graphene derivatives include graphene oxide (GO) and sulfonated graphene. GO has oxygen-containing functional groups on its surface, such as –OH and –COOH, which are easily dispersed in water and can be chemically bonded to hydration products. Therefore, the current research mainly focuses on the research of GO–cement-based materials, including cement hydration, mechanics and durability.

GO can greatly enhance the impermeability and mechanical properties of cement pastes and mortars [12,13,14,15], because GO has strong nucleation and template function, improving the microstructure of cement and enhancing mechanical properties and impermeability. Samuel Chuah [16] believes that the enhancement mechanism of GO is that GO can form a strong covalent bond with the hydration products of cement. Zhu Pan and other studies [17,18] have shown that 0.05 wt.% of the GO content increased its compressive strength by 33% and flexural strength by 59%. Wang Qin [19,20] also obtained similar results. Dong et al. [21] showed that GO can increase the rheological parameters of cement paste and reduce its fluidity. In response to this problem, the authors proposed a method of incorporating additives to improve its properties. Mohammad A Rafi [22] believes that GO–cement-based materials have strong mechanical properties and oil absorption properties, giving certain functional properties to cement-based materials. Xue guang et al. [23] compounded GO and multi-walled carbon nanotubes (SWCNTs) into cement slurries to prepare composite materials and studied their mechanical properties. The results show that 1.5 wt.% GO and 0.50 wt.% SWCNTs is the optimal dosage to improve the mechanical properties of the cement slurry. Teng Tong [24] studied the effect of GO on the mechanical properties and durability of concrete. It was found that GO can enhance its mechanical properties but its effect on the functionalities is not obvious. G.M. Kim [25] et al. studied the effect of CNT dispersion and pore structure on their electrical properties. The results show that the addition of water reducer and silica fume greatly improve its electrical properties, but their mechanism of action is different: water reducer can reduce the van der Waals force between carbon nanotubes to achieve dispersion; silica fume refines the pore size. The synergistic effect of the two, on one hand, improves the dispersion of CNTs, while on the other hand, the improvement of the pore structure helps carbon nanotubes form a conductive network in the cement matrix.

Graphene has good electromagnetic shielding properties and rGO/MWCNTs/Fe_3_O_4_ composites have very good electromagnetic shielding properties. The absorption efficiency of composite materials with only 2 mm coatings can reach 36 dB at 13.44 GHz electromagnetic wave frequency [26]. Chuangang Hu [27] showed that three-dimensional graphene–iron tetroxide nanocomposites have higher microwave absorption properties. Qiong Liu [28] showed that the mechanical properties of graphene nano-sheets and GO nano-sheets were 0.8% and 1.6%, respectively. When the graphene content is 6.4%, its piezo resistance is relatively stable. Sedaghat [29] has shown that graphene can improve the electrical conductivity and thermal diffusivity of cement materials and thus reduce the internal and external temperature differences caused by heat of hydration and weaken the structural damage caused by temperature stress.

However, the oxygen-containing functional groups on the surface of GO destroy the conjugated structure of graphene crystals and lose most of its electrical conductivity, thermal conductivity or other functions, and cannot achieve the intelligence of cement-based materials. Graphene has excellent electrical and thermal properties. Based on the above two issues, this paper intends to use GO as a functional component and blend graphene dispersion into cement-based materials to prepare cement-based functional composites. The effects of graphene on the mechanical properties, resistivity, thermal conductivity, electromagnetic shielding and pressure sensitivity of cement-based materials were studied and the mechanism was analyzed.

## 2. Experiments

### 2.1. Sample Preparation

**Figure 1:**
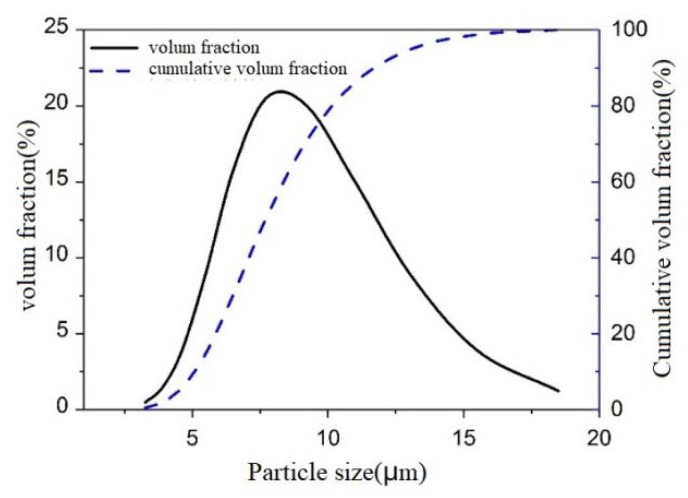
The size distribution of reduced graphene oxide (rGO).

**Figure 2:**
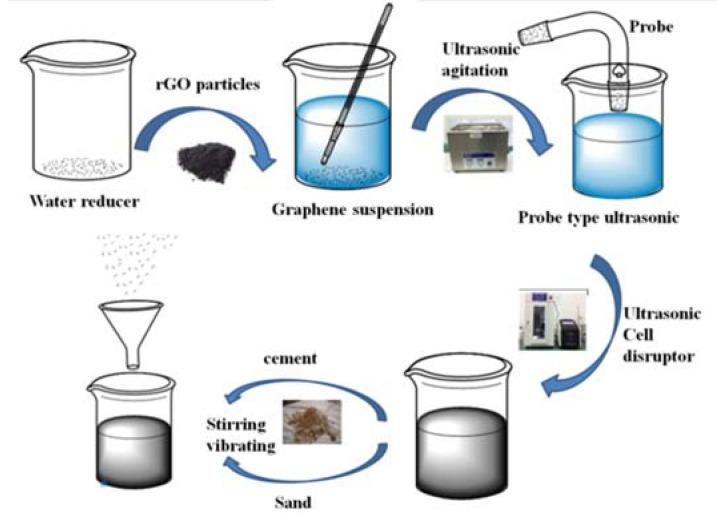
The preparation process of samples.

A commercially available P·II 52.5 cement (The Xiao Yetian Cement Co., Ltd., Nanjing, China) was used in this study and its chemical compositions are listed in Table 1. The standard sand (The Xiamen ISO Standard Sand Co., Ltd., Xiamen, China) with the fineness modulus within 2.3–3.0 was used in this study. The electrical connections were made using 20 mm × 30 mm stainless steel nets with 2 mm × 2 mm of mesh size. The physical parameters of the rGO (The Sixth Element Materials Technology Co., Ltd., Changzhou, China) are shown in Table 2. The particle size distribution of the rGO is shown in Figure 1 and the median particle size is about 8.2 μm. The naphthalene sulfonate water-reducing admixture (The Sobute Co., Ltd., Nanjing, China) was used to disperse and stabilize the rGO. A schematic map of how GNP was prepared was made and shown in Figure 2. For relatively large rGO particles, the extremely thin, but highly wrinkled surface can be observed via Transmission Electron Microscope (TEM).

The mix proportions of the samples in this study are listed in Table 3. The processes of sample preparations are in Figure 2. Firstly, 80% water is used to prepare the naphthalene water reducer. Then, put the naphthalene water reducer mixed with rGO powder in the 1500 W ultrasonic cell crusher for 60 min to obtain homogeneous dispersed rGO dispersion. Afterwards, add cement to the mortar mixer containing the rGO dispersion solutions and stir slowly for 30 s. Put the remaining 20% water and standard sand in mixer and stir slowly for 1 min, then stop for 1 min, and further stir for 4 min and prepare test specimens according to the experimental specifications. Cast the even mixed mortar to the 40 mm × 40 mm × 160 mm mold. After 24 h, the samples were demolded from the molds and placed in the curing room with 20 ± 2 °C temperature and relative humidity ≥ 95%.

### 2.2. Sample Characterization

The mechanical strength test was carried out with an electronic universal testing machine (CMT5105 Meister Company, Shenzhen, China) according to GB/T 17671-1999.

The SEM (Scanning Electron Microscope) analysis of GNP/cement composites was conducted with a FEI 3D environmental scanning electron microscopy, operating at 20 kV. The Mercury Intrusion Porosimetry (MIP) (Autopore IV 9500, Micromeritics Instrument Corporation, Norcross, GA, USA) was used to study pore structure. The pressure range is 0–113 MPa, the surface tension of mercury is 0.485 N/m, and the contact angle is 130°.The specimen with the size of 3–5 mm is cut out from the middle part of the samples and placed at 50 °C for more than 3d after immersing in the isopropyl ketone solution. The thermal conductivity is tested by the heat flow method and referenced standards ISO 8301, ASTM C518, DIN EN 12667/12939, DIN EN 13163. The 300 mm × 300 mm × 30 mm slurries are dried at 105 ℃ to constant weight and tested by a thermal conductivity tester.

**Figure 3:**
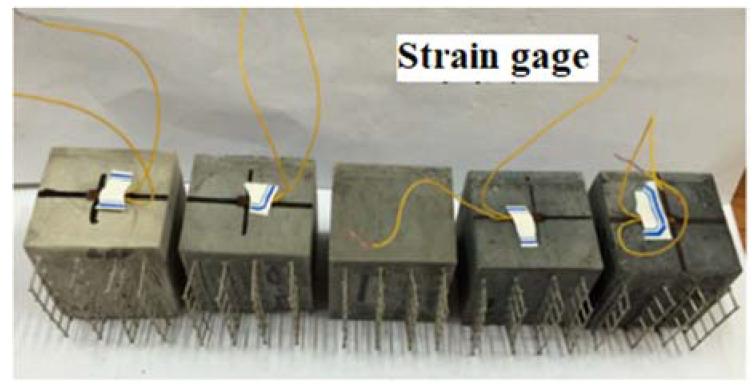
Preparation of specimen.

**Figure 4:**
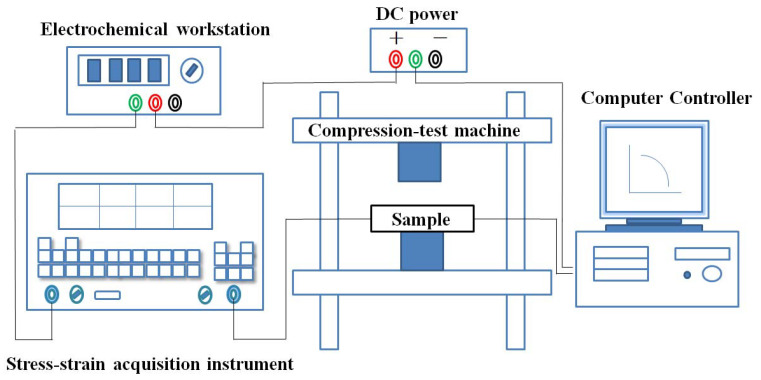
The test device of pressure sensitivity.

The size of the mortar specimen is 40 mm × 40 mm × 80 mm. The distance between the two internal electrodes is 40 mm, and the distance between the two external electrodes is 60 mm. The preparation of samples is shown in Figure 3. The DC four-electrode method was used to measure the pressure sensitive performance and the electronic universal testing machine (CMT5105) was used as the loading device of the test. The external electrode of the test piece is connected with 15 V DC voltage, the electrochemical workstation is used to record the voltage across the electrode in the test piece during the loading process in real time. The micro-strain in the loading direction of the test piece was recorded continuously using a digital acquisition device. The experimental devices are shown in Figure 4. The voltage is set 15 V [30]. Use a constant speed 50 N/s to 8 kN, then reduce to 0 kN, which was a complete loading–unloading cycle and repeated 6 times. The electrochemical workstation collects the electrode voltage in the test piece every 0.1 s and the total time is 4000 s. The data of micro-strain collected by stress–strain acquisition instrument is recorded every 1 s.

## 3. Results and Discussion

### 3.1. Effect of rGO on Mechanical Property of Cement Mortars

**Figure 5:**
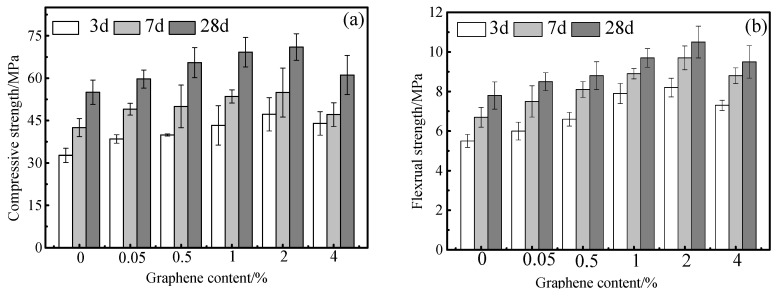
The mechanical properties of cement mortar with different ages and rGO content, (**a**) Compressive strength, (**b**) Flexural strength.

Figure 5 shows the mechanical properties of cement mortar specimens of different rGO content. Figure 5a shows the changes of compressive strength. It can be seen that the compressive strength increased first and then decreased with the increase of rGO. When the rGO content is 2.00 wt.%, the compressive strength at different hydration ages reached their maximum value. In addition, the effect of rGO is more obvious on the early compressive strength and 2.00 wt.% rGO increased 44% compressive strength for samples cured 3d. Figure 5b shows that the flexural strength of specimens in different ages is consistent with their compressive strength. Similarly, the effect of rGO on the early flexural strength was more obvious and the flexural strength of mortar with 2.00 wt.% rGO cured for 3 d increased by 49%.

Table 4 shows the mechanical properties of cement mortar specimens with different rGO content for 28 d. It can be seen from the table that the compressive and flexural strength of cement mortar was improved after the incorporation of rGO. Compared with the control group, the compressive strength of sample GM2.00 increased 29% and reached the maximum value of 71.0 MPa. However, the sample GM4.00 only increased 11% with higher rGO content, indicating that the excessive amounts of rGO are not conductive to the increase in compressive strength, which may be possibly due to agglomeration of rGO. The changing trend of the flexural strength is similar to that of the compressive strength. The flexural strength of GM2.00 reached the maximum value 10.5 MPa, which is 35% higher than that of the blank.

### 3.2. Effect of rGO on Pore Structure of Cement Mortar

**Figure 6:**
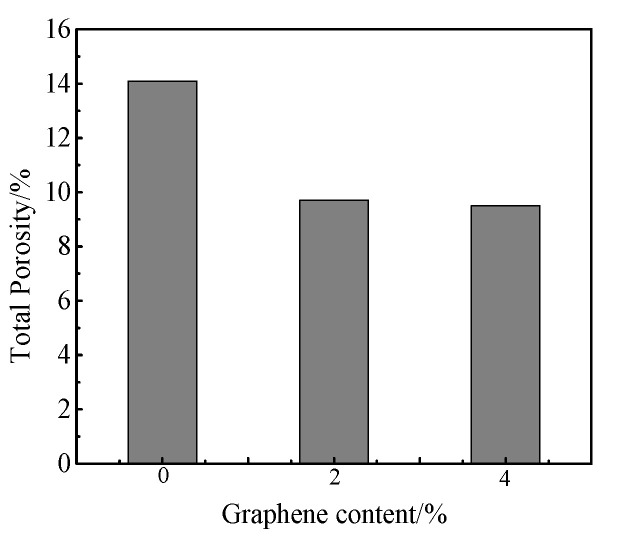
The porosity of cement mortar with different rGO content.

**Figure 7:**
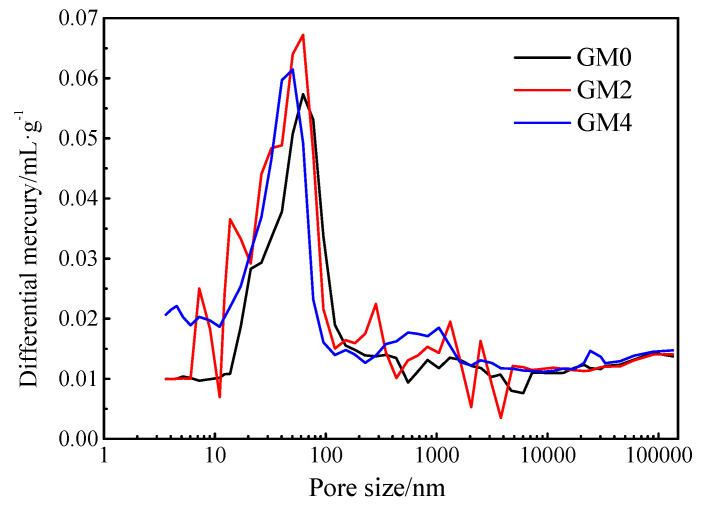
The pore size distribution pf slurry with different rGO content.

Figure 6 shows the porosities of cement mortar decreased with the decreasing addition of rGO. Compared with the base sample, the porosity reduced 31% when the rGO content is 2.00 wt.%. However, the porosity of mortar prepared with 4.00 wt.% rGO reduced just 33%. On one hand, this indicates that the ability to disperse rGO is limited due to the easy agglomeration between rGO particles. It is hard to disperse uniformly when rGO is over 2.00 wt.%. On the other hand, the optimum amount of rGO was 2.00 wt.%. Figure 7 is a pore size distribution of slurry with different rGO content. It can be seen that with the increasing rGO content, the number of small pores first increases and then decreases. The number of macrospores is nearly the same. This indicates that graphene has a plugging effect and the appropriate amount of rGO increases the number of small pores. However, the extensive rGO leads to agglomeration, which no longer improves the microstructure of the slurry. This trend is consistent with the strength change. The results in Table 5 show that with the increasing rGO content, the average pore size, the median pore size and the most probable pore size appear in a decreasing trend, which indicates the rGO refines the pore structure and enhances its mechanical properties.

### 3.3. Functional Properties of rGO–Cement Composite Materials

#### 3.3.1. Electrical Conductivity and Thermal Conductivity

**Figure 8:**
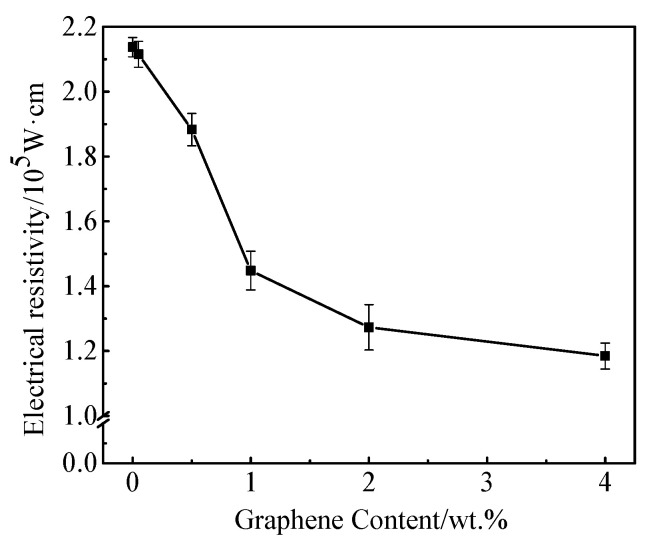
The relationship between electrical resistivity and rGO content.

The seepage theory is that the composite resistivity does not always change in proportion with the amount of conductive particles. When the conductive particles increase to a certain critical, the resistivity changes significantly and then slowly. The critical value is referred as a threshold. Figure 8 shows the changes of resistivity of mortar with different rGO content.

It can be seen from Figure 8 that the resistivity decreases firstly and then stabilizes with the increasing content of rGO. When rGO content changes from 0.50 wt.% to 2.00 wt.%, the resistivity decreases drastically. Compared with the base sample, the resistivity of cement mortar with 2.00 wt.% decreased 40%. If rGO is continuously added, the change in resistivity is no longer obvious. Therefore, the threshold value of rGO is around 2.00 wt.%. When the rGO content is 4.00wt.%, the resistivity decreased 45%, from 2.137 × 10^5^ Ω·cm to 1.185 × 10^5^ Ω·cm.

The reason for the phenomenon above may be that the rGO did not form a conductive network inside the cement matrix when the content is under 2.00 wt.%. The resistivity change is not obvious. When the rGO content is 2.00 wt.%, a certain conductive network begins to form inside the cement matrix and the electrons easily migrate, which leads to the sharp drop of the resistivity. When the rGO exceeds 2.00 wt.%, the internal conductive networks slowly laps and the resistivity slowly decreases. The measured density of different cement containing rGO is shown in Table 6.

**Figure 9:**
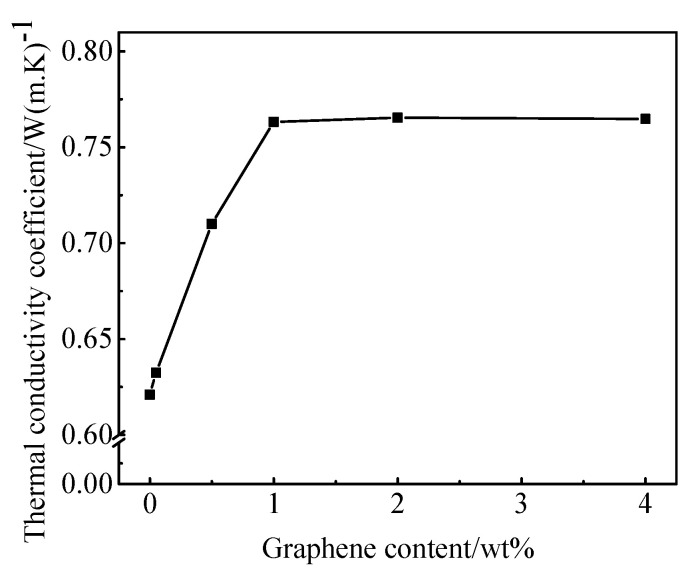
The thermal conductivity coefficient of different cement mortar.

Figure 9 shows that the thermal conductivity coefficient of mortar mixes increases first and then stabilizes with the increasing rGO content. When 1.00 wt.% rGO is added, the thermal conductivity of cement mortar is 0.77 W/(m·K), which is 23% larger than the control sample. But when rGO was continuously added, the thermal conductivity leveled off.

#### 3.3.2. Electromagnetic Shielding Property

The studies above show that 1.00 wt.% rGO can significantly improve the electric and thermal conductivity of cement-based composites. This experiment used the waveguide method to study the electric parameters of paste with 1.00 wt.% rGO to characterize the electromagnetic shielding properties and analyze the influence of rGO on the electromagnetic shielding performance.

**Figure 10:**
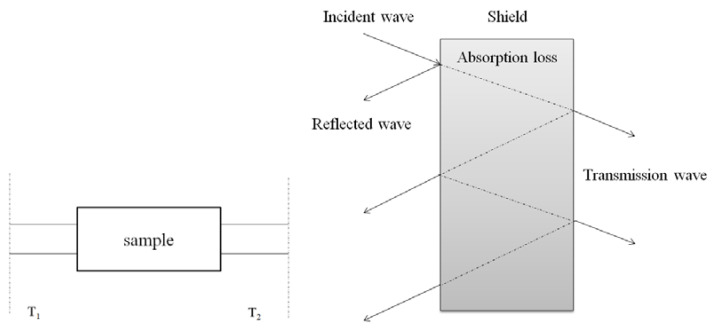
The schematic analysis of electromagnetic shielding mechanism.

It can be seen from Figure 10 that the electromagnetic wave shielding mainly includes three parts: reflection loss on the shield surface, internal absorption loss and multiple reflection loss. In Figure 10, T_1_ and T_2_ are the input reference plane and the output plane, respectively. All measured parameters are relative to the reference plane.

According to vector network theory, the scattering matrix of a two-port network is:

(1) $$\left[S\right]=\left(\begin{array}{cc} S_{11} & S_{12}\\ S_{21} & S_{22} \end{array}\right)$$

where T1 and T2 are the input reference plane and the output plane, respectively. S_11_—Reflection coefficient on the T1 surface when T2 is connected to the matched load; S_12_—Reverse transmission coefficient from T2 to T1 when T2 is connected to the matched load; S_21_—Reverse transmission coefficient from T1 to T2 when T1 is connected to the matched load; S_22_—The reflection coefficient on the T2 surface when T1 is connected to the matched load.

For materials, the equivalent network is reciprocal, so by definition, when the end of the network is matched to the load, the transmission coefficient of the material is T = S_21_ = S_12_, and thus the shielding effectiveness is SE = 20lg$\frac{1}{\left|T\right|}=20\mathrm{lg}\frac{1}{\left|S_{12}\right|}$.

**Figure 11:**
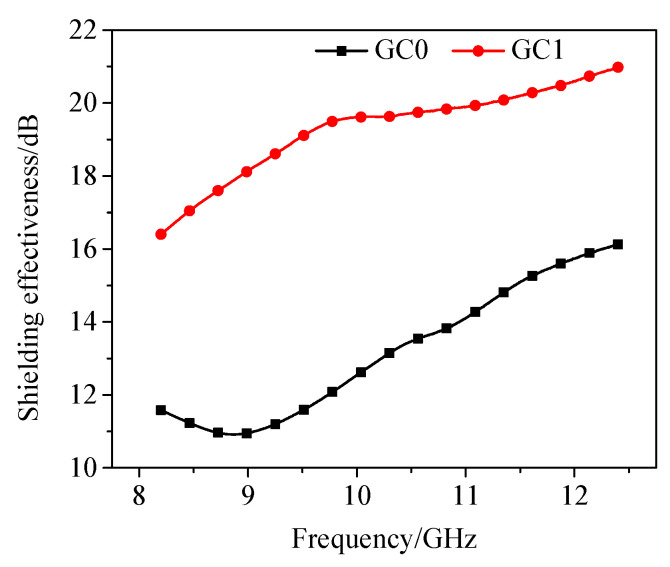
The shielding effectiveness of cement paste with rGO.

It is found from Figure 11 that the shielding effectiveness of the blank paste is 11–16 dB when the electromagnetic wave frequency is between 8.2–12.4 GHz, which is considered to have a weak electromagnetic shielding effect. After 1.00 wt.% rGO is incorporated, the shielding effectiveness increases from 30% to 45%, reaching 16–21 dB. In addition, as the frequency increases, the shielding effectiveness gradually increases.

Due to the mating force between rGO and cement base, the rGO molecules connect a good interface with each other to form a conductivity network, which is favorable for transporting carriers and thus canceling the incident electromagnetic field.

**Figure 12:**
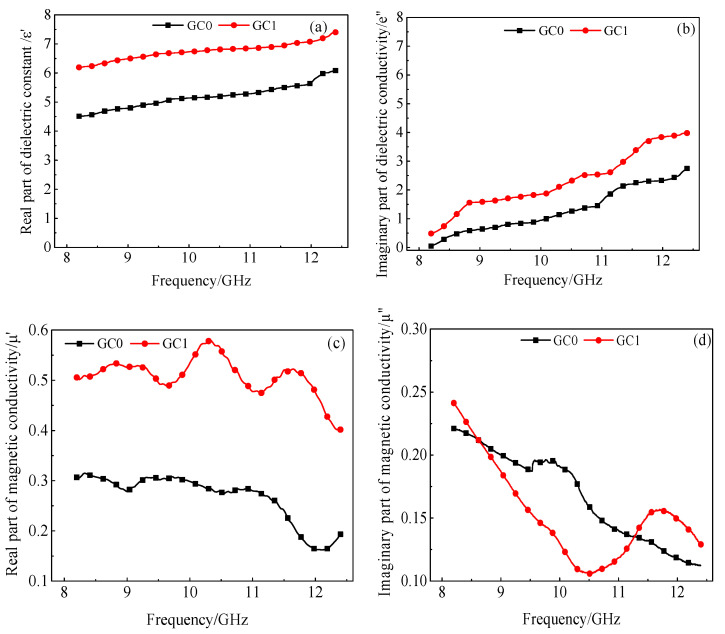
The electromagnetic parameters of cement paste with different rGO. (**a**) real part of dielectric conductivity (**b**) imaginary part of dielectric conductivity; (**c**) real part of magnetic conductivity (**d**) imaginary part of maginary conductivity.

In Figure 12, ε’ represents the real part of the complex permittivity, indicating the polarization degree of the material under the electric field; ε’’ is the imaginary part of the complex permittivity and is the loss measurement caused by the rearrangement of the electric dipole moment of the material under the electric field. μ’ represents the real part of the complex magnetic μ_r_, indicating the polarization degree of the material under the magnetic field; μ’’ is the imaginary part of the complex magnetic and is the loss measurement caused by the rearrangement of magnetic dipole moment of the material under the magnetic field. The imaginary part can reflect the ability of the material to lose electromagnetic waves. The larger the imaginary part is, the greater the loss of electromagnetic waves from the material.

It is shown from Figure 11 that the real part of the dielectric constant of the blank cement paste is 4.5–6, the incorporation of 1.00 wt.% rGO made the real part value increase 38%, which indicates that the dielectric polarization degree of the cement paste sample increases under the electric field. Meanwhile, the incorporation of 1.00 wt.% rGO increased the imaginary part of the dielectric constant of cement paste specimen by 60%, which indicates that rGO increases the dielectric loss of the composites to electromagnetic waves. However, the values of the real and imaginary parts of the permeability of the slurry and rGO–cement paste specimens are close to zero, which may be due to the lack of magnetic loss capability of rGO.

#### 3.3.3. Pressure Sensitive Performance

##### Cement Paste with Different rGO Content

**Figure 13:**
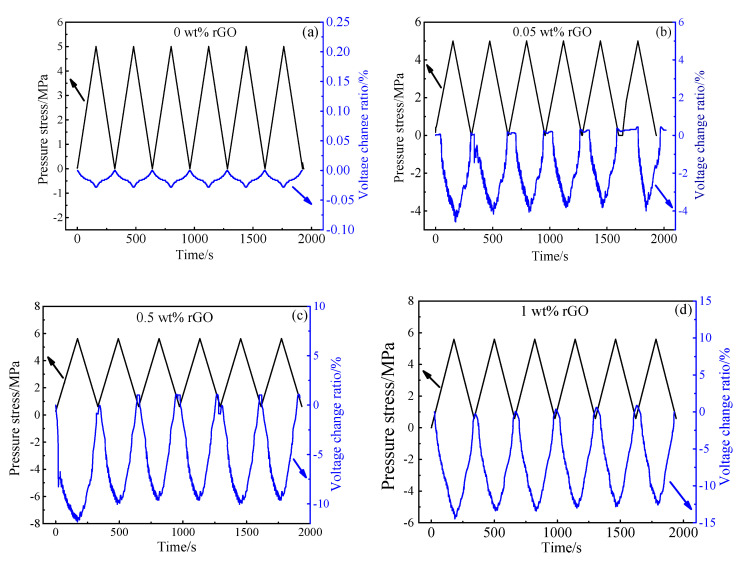

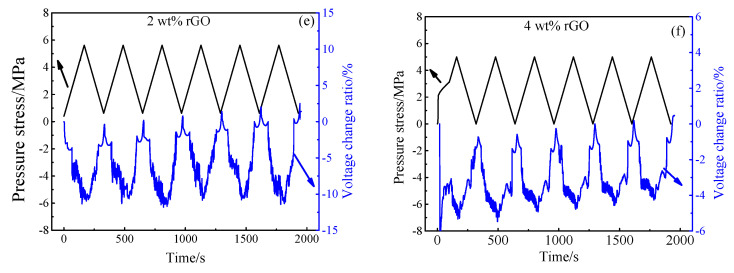
Voltage change ratio and pressure stress of different rGO–pastes under six-cycle loading (**a**) 0 wt.% rGO (**b**) 0.05 wt.% rGO (**c**) 0.5 wt.% rGO (**d**) 1 wt.% rGO (**e**) 2 wt.% rGO (**f**) 4 wt.% rGO.

**Figure 14:**
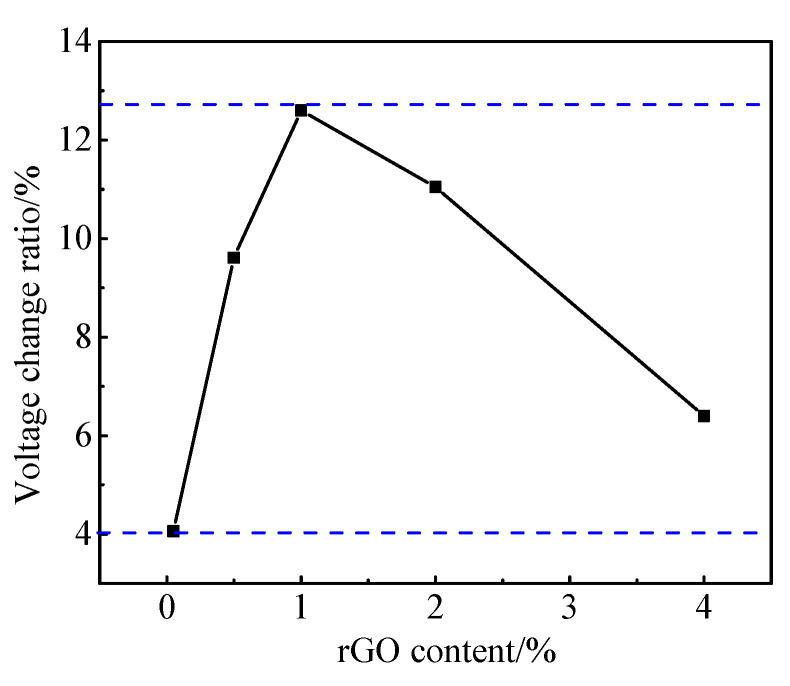
The relationship between maximum voltage change ratio and rGO content.

Figure 13 and Figure 14 show the voltage rate changes of the cement paste with 0.00–4.00 wt.% rGO at six cycles of loading. It can be seen from the figure that there must be a relationship between the voltage change and the load stress. With the increasing rGO content, the linearity and signal-to-noise ratio of this relationship first increased and then decreased. During the loading process, the voltage of samples trended to decrease monotonously; While during the unloading process, the voltage had a trend of monotonous increase. Therefore, the appropriate amount of rGO is beneficial to enhance the stability and pressure sensitive property. The pressure sensitivity of GC0.5 and GC1 samples was better than that of the lower dosages of GC0.05 and the higher dosages of GC2 and GC4. The linearity and signal-to-noise ratio showed similar patterns. Therefore, 0.50–1.00 wt.% rGO is the optimum range of rGO content for pressure sensitivity.

It can be seen from Figure 12a that the voltage of cement paste remained almost the same under the six-cycle loading. It shows that cement paste does not have a pressure sensitivity without rGO. As shown in Figure 13b, when the applied load is 5 MPa, the voltage change rate of the paste with 0.05 wt.% rGO is 4.06%. Figure 13c,f show that when the load reaches 5 MPa, the voltage change ratio is 6.4–12.6% when rGO content is 0.50–4.00 wt.%. It can be seen from Figure 13g that the pressure sensitivity of paste with 0.00–4.00 wt% rGO increased first and then decreased. Among these, the pressure sensitivity of paste with 1.00 wt.% rGO was the most obvious and the voltage change rate reached 12.6%.

When rGO content is 0.50–1.00 wt.%, the change rate of samples has a similar changing trend as the synchronous loading during the whole cycle. It can be inferred that the pressure sensitiveness of samples in the elastic region is superior and the voltage change can also reflect the external stress change. Each voltage change value of samples corresponds to each cyclic stress value. In the first loading cycle of the sample, the slight irreversible increase can be found in voltage and then the voltage change tends to be stable. This result corresponds to the findings of Sasmal Saptarshi.

**Figure 15:**
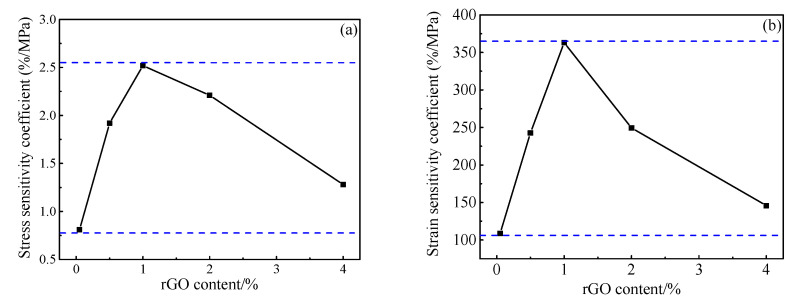
Stress and strain sensitivity coefficients of pastes with different rGO content (**a**) stress (**b**) strain.

Figure 15 shows the stress and strain sensitivity coefficients of the specimens with different rGO contents. It can be seen that the stress and strain sensitivity coefficients of the specimens tend to increase gradually and then slowly decrease with the increasing rGO. When the amount of rGO is 1.00 wt.%, the stress sensitivity is as high as 2.52%/MPa, the strain sensitivity is 363.10 and the voltage change is the most obvious. Thus the test piece pressure sensitivity is optimal.

##### Mortar with Different rGO Content

Due to the large particle size of the sand, it will block the interrelationship of rGO in the cement matrix, making it difficult to form a conductive network. Under the same rGO content, the pressure sensitivity of the rGO–mortar specimen may be worse than that of rGO–paste. However, the mortar volume stability and hydration heat are superior to the paste. In order to investigate the pressure-sensitive properties of mortar materials, mortar composite materials with 0.00–4.00 wt.% rGO content were prepared, and the voltage–stress response relationship of rGO–mortar specimens was tested to study the elastic range of different rGO–mortar specimens.

The rGO–cement mortar specimen size is 40 mm × 40 mm × 80 mm, and its pressure sensitivity is tested by the DC four-electrode method at 50 N/s loading rate. The experimental results are shown in the figures below.

**Figure 16:**
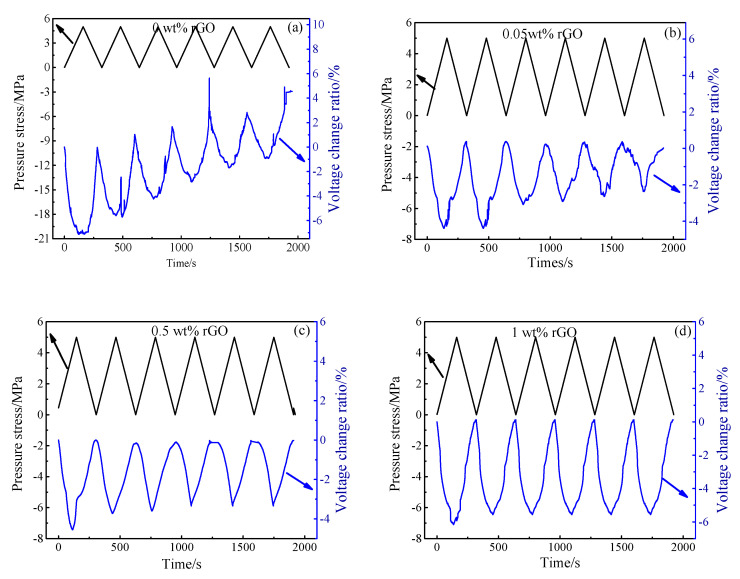

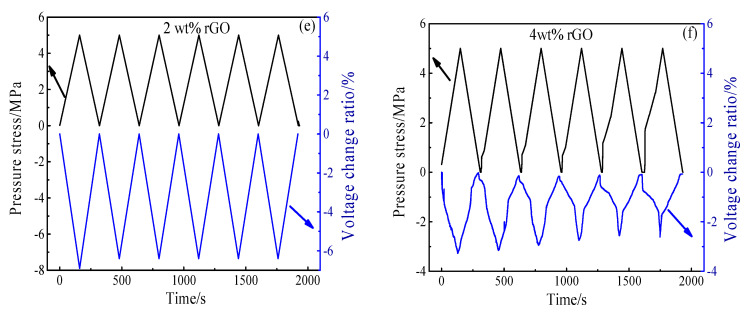
The voltage change ratio and pressure stress of different rGO–mortars under six-cycle loading (**a**) 0 wt.% rGO, (**b**) 0.05 wt.% rGO, (**c**) 0.5 wt.% rGO, (**d**) 1.0 wt.% rGO, (**e**) 2.0 wt.% rGO, (**f**) 4.0 wt.% rGO.

Figure 16 shows the voltage change rate of 0.00–4.00 wt.% rGO–mortar specimen under six cycles of loading. It can be seen from Figure 15 that the pressure sensitivity of the rGO–mortar test piece is slightly different from that of the rGO–paste at the same loading speed and loading amplitude. When the rGO content is 0.50 wt.%, the pressure sensitivity stability of the rGO–mortar samples is worse than that of the rGO–paste. When the content of rGO reaches 1.00 wt.%, the pressure sensitivity of rGO–cement paste reaches the optimized value 12.6%, but the voltage change rate of rGO–mortar is just 5.13%. When rGO content reached 2.00 wt.%, the pressure sensitivity of the rGO–mortar was optimized, but the value was only 6.4%. This shows that the pressure sensitivity is greatly reduced after the introduction of fine aggregate sand.

**Figure 17:**
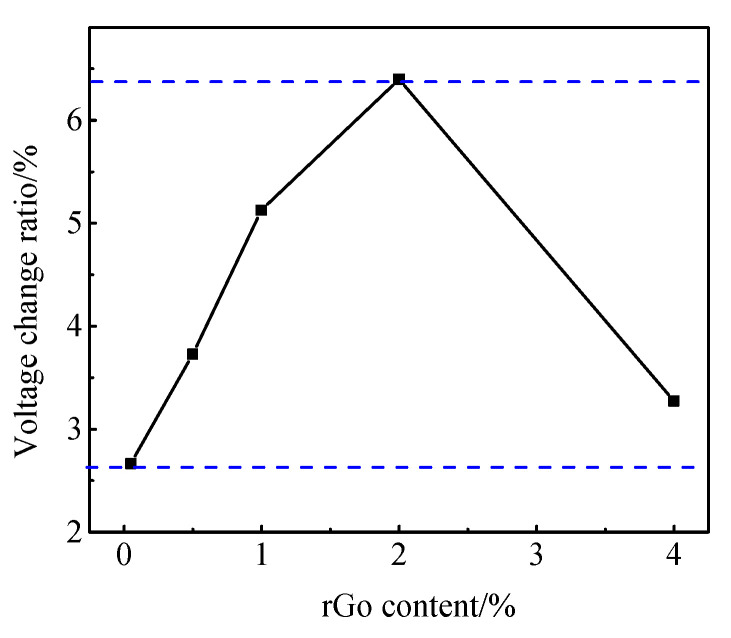
The relationship between the voltage change ratio and rGO content.

**Figure 18:**
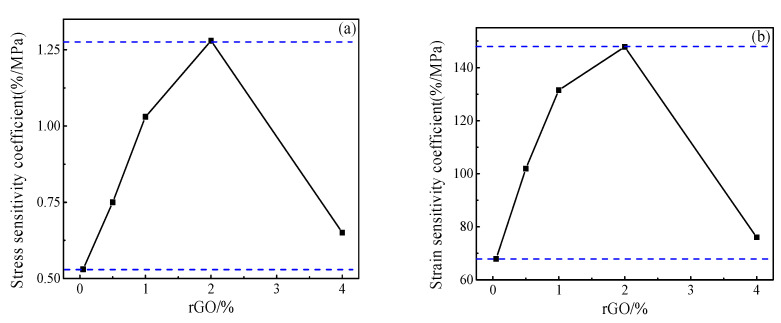
The stress and strain sensitivity coefficients of different rGO–mortars (**a**) stress (**b**) strain.

Figure 17 and Figure 18 show the stress and strain sensitivity coefficients of mortar specimens with different rGO content. It can be seen that the stress and strain sensitivity coefficients of the specimen also increase first and then decrease with the increasing rGO. The difference is that when the rGO content is 2.00 wt.%, the stress sensitivity coefficient is as high as 1.28%/MPa, and the strain sensitivity coefficient is 147.80. Compared with the maximum stress and strain sensitivity coefficient of the paste specimens of 2.52%/MPa and 363.10, the values of the mortar specimens were reduced by 49% and 59%, respectively.

##### The Mechanism of Pressure Sensitive Properties of rGO Composites

**Figure 19:**
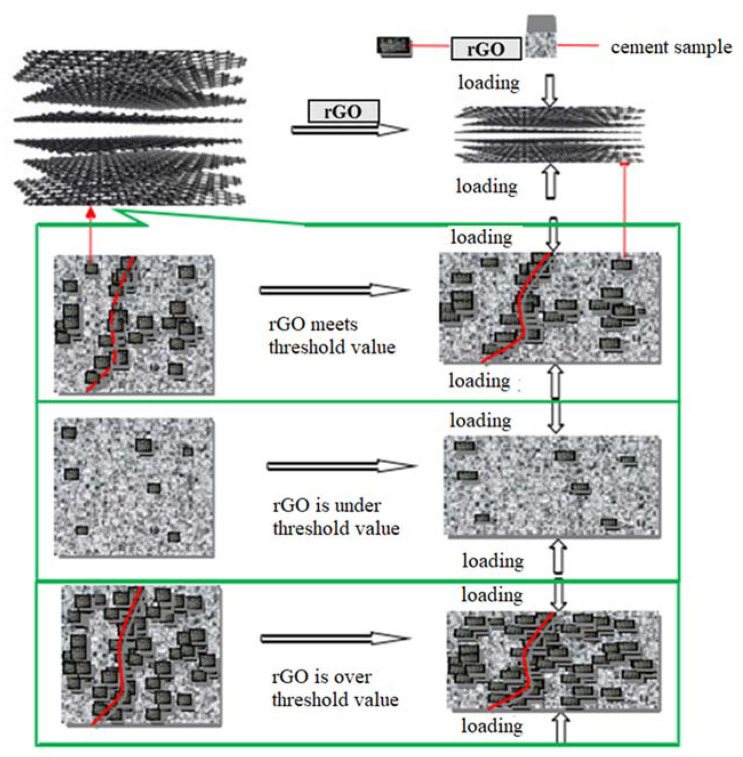
The schematic diagram of pressure sensitive performance of rGO composites.

It can be seen from Figure 19 that the distribution of rGO in the cement changes with the loading and unloading process. When the rGO content is under the threshold, rGO particles are far apart from each other. Therefore, the 0.05 wt.% rGO–cement composites has a poor pressure sensitivity stability.

When the rGO content reaches the threshold, a conductive network begins to form in the cement matrix. If the specimen is deformed when subjected to compressive stress, the distance between the rGO particles begins to decrease, making it easier for electrons to migrate in the cement matrix, as shown in Figure 19. Therefore, rGO–cement composites exhibit stable and excellent pressure-sensitive properties under pressurized conditions [31,32].

When the amount of rGO is excessive, the rGO particles are easily agglomerated, so the pressure sensitivity of the test piece is lowered. In the mortar, due to the presence of fine aggregate sand, more rGO particles are needed to form a conductive network. In the paste test piece, when the rGO content exceeds 1.00 wt.%, the pressure sensitivity of the test piece begins to decrease; while for the mortar samples, when the rGO content exceeds 2.00 wt.%, The pressure sensitivity of the test piece began to decline. Therefore, the optimum pressure sensitivity of the paste and mortar samples corresponds to a rGO blending amount of 1.00 wt.% and 2.00 wt.%, respectively.

**Figure 20:**
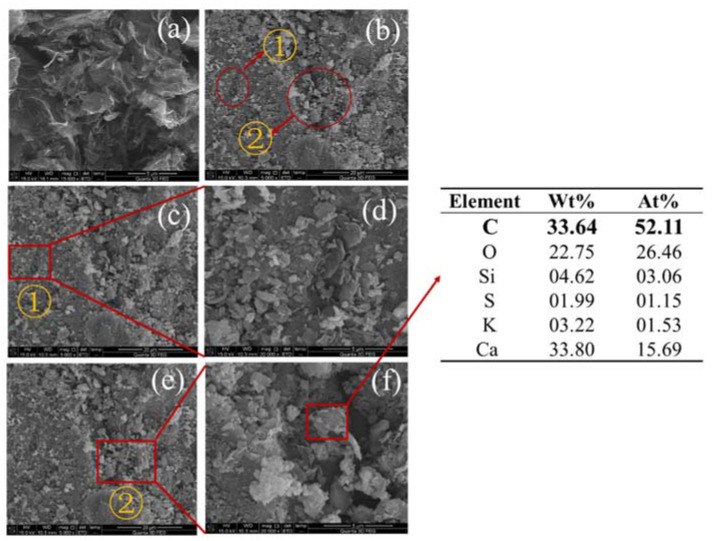
SEM photographs of cement paste with 1.00 wt.% rGO (**a**) rGO (**b**) paste (**c**) area ① (**d**) magnifying view of area ① (**e**) area ② (**f**) magnifying view of area ②.

**Figure 21:**
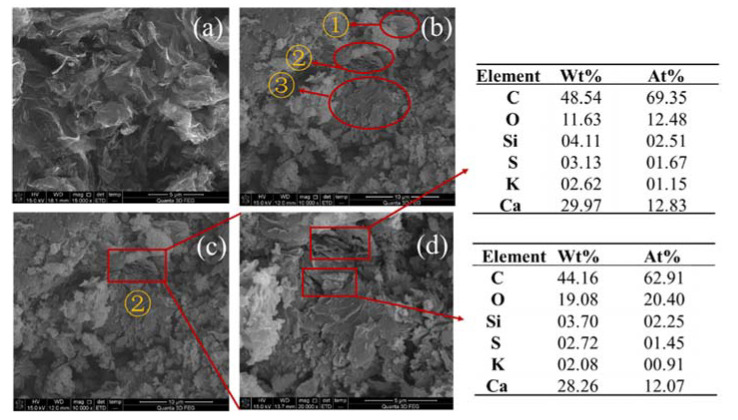
SEM photographs of cement paste with 4.00 wt.% rGO, (**a**) rGO (**b**) paste (**c**) area ② (**d**) magnified view of area ②.

It can be seen from the Figure 20 and Figure 21 that when the rGO is 1.00 wt.% and 4.00 wt.%, a conductive network has been formed inside the cement paste, and electrons are easier due to tunneling effects and contact effects [33,34]. Migration may also be the reason for its improved functionality. When the amount of rGO is 1.00 wt.%, electron percolation is generated in the cement paste, and the percolation threshold is reached. When the amount of rGO is 4.00 wt.%, the distance between the rGO particles is closer, and even contact with each other, and more agglomeration occurs, and the functional improvement effect is not obvious.

After the incorporation of rGO, the thermal conductivity of the cement mortar would be improved, probably due to the good channel formed by rGO inside the cement. The enhancement mechanism of the shielding performance of rGO cement paste composites mainly includes the following aspects:

(1) rGO has excellent electrical conductivity, which makes rGO exhibit strong dielectric loss capability for electromagnetic waves. Thus, it increases the shielding performance of paste to the electromagnetic wave.
(2) In addition, the two-dimensional laminated structure of rGO increases the reflection area of electromagnetic waves, so that the single reflection loss and shielding effectiveness of the electromagnetic wave of the rGO–cement paste is enhanced.

## 4. Conclusions

In this paper, rGO is introduced in cement-based materials as a reinforcing component and a functional component to prepare rGO–cement-based composite materials. Using different analysis and testing methods to study the mechanical properties, conductivity, thermal conductivity, electromagnetic shielding and pressure sensitivity of rGO–cement-based composites, the following conclusions were obtained:

(1) The incorporation of rGO can generally enhance the mechanical properties of rGO–cement mortar, and the increase of early strength of cement mortar is more obvious. The optimum content is 2.00 wt.% rGO, which increased the 3 d compressive strength and flexural strength of cement mortar specimens by 44% and 49%, respectively.
(2) With the increasing rGO content, the electric conductivity and thermal conductivity of rGO–cement mortar composites increase first and then stabilize. When the amount of rGO is 2.00 wt.%, the resistivity of the test piece is basically stable, which is reduced 40%, from 2.14 × 10^5^ Ω·cm to 1.27 × 10^5^ Ω·cm. However, when the rGO content was 1.00 wt.%, the thermal conductivity was stable at 0.77 W/(m·K), which was 23% higher than the blank group.
(3) rGO improved the dielectric polarization of the rGO–cement paste specimen under the electric field. The results from the vector network analyzer show that 1.00 wt.% rGO increases the imaginary part of the dielectric constant of cement paste by 60% and increases the real part of the dielectric constant of the cement paste composite by 38%, which indicates that rGO significantly increases the dielectric loss of cement paste.
(4) The relationship between the strain–stress sensitivity coefficient and rGO content was studied. As the amount of rGO increased, its pressure sensitivity showed a tendency to rise first and then decrease. For the paste composite, when the rGO content is 1.00 wt.%, the pressure sensitivity has a maximum stress 2.52%/MPa and a strain sensitivity of 363.10; for the mortar composite, when the rGO content is 2.00 wt.%, the pressure sensitivity has a maximum stress of 1.28%/MPa and a strain sensitivity of 147.80, which indicates that slurry has better pressure sensitivity than mortar material.

## Figures and Tables

**Table 1:** Physical and chemical parameters of cement.

NO.	CaO	SiO_2_	Al_2_O_3_	Fe_2_O_3_	SO_3_	MgO	Loss	Specific Surface Area/m^2^kg^−1^
P·Ⅱ	64.85	21.65	5.56	4.32	2.58	0.84	1.27	350

**Table 2:** The physical parameters of rGO.

Specific Surface Area/m^2^g^−1^	Thickness/nm	Particle Size/μm	Carbon Content/%	Electric Conductivity/Sm^−1^
225.5	<5	7.06	>98.0	5352

**Table 3:** The mix of cement samples.

Sample	Water/Cement	Binder/Sand	Admixture/wt.%	
			rGO/%	Water Reducer/%
* GC0	0.45	-	0.00	0.00
GC0.05		-	0.05	0.15
GC0.5		-	0.50	0.30
GC1		-	1.00	0.50
GC2		-	2.00	1.00
GC4		-	4.00	2.00
* GM0		1:3	0.00	0.00
GM0.05		1:3	0.05	0.15
GM0.5		1:3	0.50	0.35
GM1		1:3	1.00	0.50
GM2		1:3	2.00	1.00
GM4		1:3	4.00	2.00

* GC = cement paste with rGO; GM = cement mortar with rGO.

**Table 4:** Properties of cement mortar with different rGO content for 28 d.

Sample	rGO Content/%	Compressive Strength/MPa	Standard Deviation	Flexural Strength/MPa	Standard Deviation	Compressive Strength Increase/%	Flexural Strength Increase%
GM0.00	0.00	55.0	4.30	7.8	0.69	-	-
GM0.05	0.05	59.7	3.20	8.5	0.45	9	9
GM0.50	0.50	65.5	5.33	8.8	0.70	19	13
GM1.00	1.00	69.2	5.21	9.7	0.47	26	24
GM2.00	2.00	71.0	4.71	10.5	0.80	29	35
GM4.00	4.00	61.1	6.95	9.5	0.82	11	22

**Table 5:** Characteristic pore size of slurry with different rGO content.

Sample	Average Pore Size/nm	Median Pore Size/nm	Maximum Pore Size/nm
GM0	54.4	63.3	64.3
GM2	35.2	51.2	59.4
GM4	25.9	46.4	48.3

**Table 6:** Measured density of cement mortar specimens with different rGO content.

Sample	PM	GM0.05	GM0.5	GM1	GM2	GM4
Density/kg·m^−3^	2110.3	2164.3	2113.1	2205.6	2254.1	2257.6
